# Nodular scleroderma partially controlled with tofacitinib^☆^

**DOI:** 10.1016/j.abd.2024.02.003

**Published:** 2024-08-05

**Authors:** Kai-Yi Zhou, Qian Ye, Sheng Fang

**Affiliations:** a Department of Dermatology, The First Affiliated Hospital of Chongqing Medical University, Chongqing, China; b Department of Dermatology, Plastic Surgery Hospital, Chinese Academy of Medical Sciences, Peking Union Medical College, Beijing, China

Dear Editor,

A 48-year-old man presented to the dermatology clinic with a 1-year history of systemic sclerosis (SSc) and was given a low-dose steroid of 20 mg prednisone orally per day for treatment of the disease. He presented clinically with diffuse skin hardening and tightening, as well as Raynaud's phenomenon and fulfilled the diagnostic criteria of the ACR criteria. Three months ago, he developed multiple indurated and exophytic nodules over the trunk, scattered over the lower and upper extremities, with sparing of the face. The lesions became progressively harder and more numerous and increased in size. The physical examinations demonstrated multiple, firm, raised, skin-colored nodules on the chest, abdomen, and back, and scattered over extremities (Fig. 1). The laboratory tests revealed an elevated erythrocite sedimentation rate (ESR, ESR 46 mm/1 h). He had a positive antinuclear antibody (titer 1:640) and SCL70 antibodies with a decreased serum complement Component 3 (C3, 0.78 g/L), while anti-Smith autoantibody titers were at a normal level. Cardiovascular, respiratory and esophagus examinations were unremarkable. His liver and kidney functions were normal and there were no signs of scleroderma renal crisis. The clinical diagnosis of nodular scleroderma was corroborated by a biopsy specimen that revealed a proliferation of myofibroblasts and thickened sclerotic collagen bundles in the dermis (Fig. 2A–B). Besides, the van Gieson stain showed preservation of elastic fibers, while the crystal violet stain was negative.

**Fig. 1:**
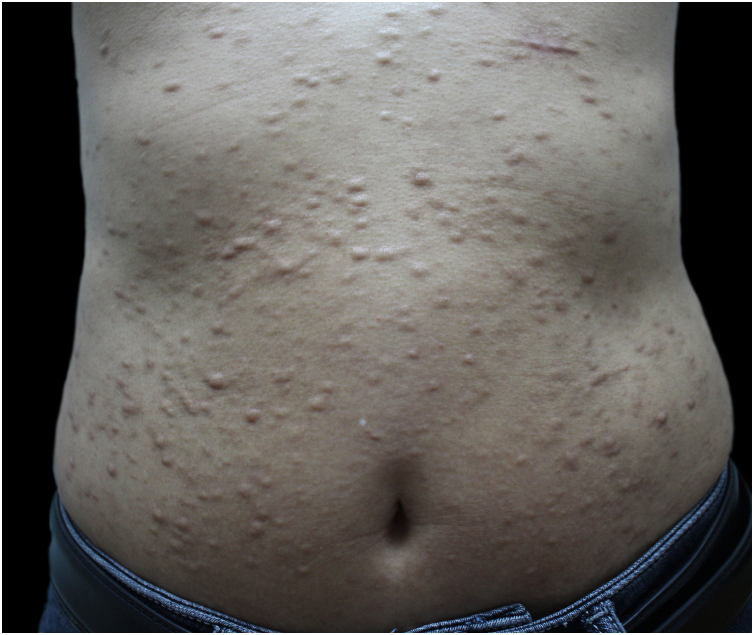
Clinical presentation before treatment with tofacitinib.

**Fig. 2:**
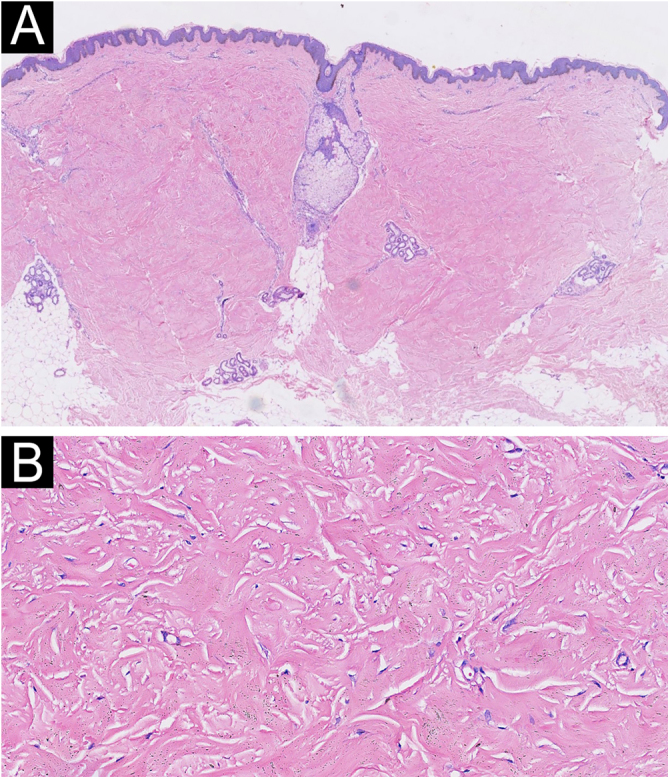
Histopathology revealed a proliferation of myofibroblasts and thickened sclerotic collagen bundles in the dermis (A, Hematoxylin & eosin ×20; B, Hematoxylin & eosin ×200).

Initial treatment with methotrexate at a dose of 15 mg/week was discontinued after three months, as the disease progressed, and new nodules appeared. Thereafter, mycophenolate mofetil at a dosage of 1 g twice daily was started. Three months later, the patient still had no observable effect. Thus, he was switched from prior treatment to tofacitinib. At the outpatient follow-up, the patient had not developed new lesions, while the pre-existing nodules were found to be smaller and less firm with continuous effects after eight months of treatment with tofacitinib (Fig. 3). He is still being followed up and has not experienced any adverse side effects.

**Fig. 3:**
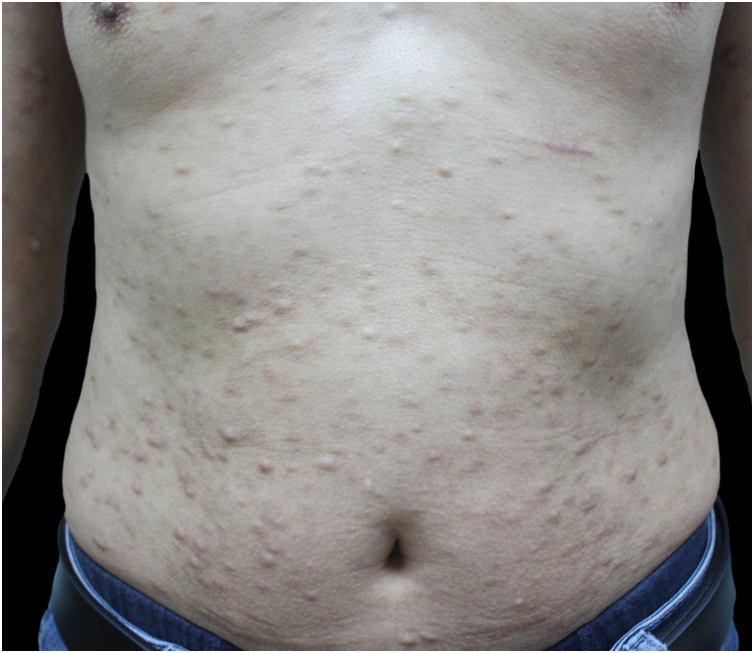
Continuous effects after eight months of treatment with tofacitinib.

Nodular scleroderma, also described as keloidal scleroderma, is a rare variant of scleroderma, which was first reported by Addison in 1854.^1^ The underlying etiological mechanisms of nodular scleroderma are still being elucidated. However, cutaneous lesions are common in patients with progressive SSc. The JAK/STAT pathway is involved in some of the major mediators implicated in the pathogenesis of SSc: IL-6, IFN type 1 and 2, IL-4 and IL-13.^2^ The pan-JAK inhibitor tofacitinib has the ability to inhibit JAK1, JAK2 and JAK3 and the downstream signaling of several cytokines. Earlier evidence also showed that patients with SSc had increased IL-4 and IL-13-activated effector B cells, promoting fibrosis.^3^

Notably, it was shown that JAK/STAT inhibitors attenuate fibrosis in the skin of different mouse models.^4^ Our patient's favorable outcome suggests that tofacitinib may be an effective option for treating nodular scleroderma. Although our study was consistent with other studies, further studies are recommended to elucidate the role of JAK inhibitors in nodular scleroderma. Off-label treatment dominates the clinical management of rare diseases. In this article, the repurposing of tofacitinib in nodular scleroderma with a long-term follow-up is presented.

## Financial support

None declared.

## Authors’ contributions

Kai-Yi Zhou: Collection, analysis, and interpretation of data; Drafting and editing of the manuscript; Critical review of the literature.

Qian Ye: Collection, analysis, and interpretation of data; Drafting and editing of the manuscript; Critical review of the literature.

Sheng Fang: Design and planning of the study; Editing and final approval of the manuscript.

## Conflicts of interest

None declared.
